# Supplementary material for: MetaRibo-Seq measures translation in microbiomes
Source: Nat Commun. 2020 Jun 29;11:3268. doi: 10.1038/s41467-020-17081-z (PMC7324362; doi:10.1038/s41467-020-17081-z)
Supplement: Supplementary file 10 — Supplementary Data 7 [file 41467_2020_17081_MOESM10_ESM.zip › File2/Confidence_High_Taxonomy/108575_out.krona.html]

Javascript must be enabled to view this page.

members
magnitude
magnitudeUnassigned
count
unassigned
taxon
rank

108575\_out

68

68
superkingdom
2

68
phylum
1239

class
67
186801

order
67
186802

541000
67
family


SRS017859\_contig\_number\_contig-100\_10655.10656SRS050026\_contig\_number\_contig-100\_16519.52858SRS053356\_contig\_number\_contig-100\_7702.43995SRS075878\_contig\_number\_24107SRS100907\_contig\_number\_contig-100\_38698.38699SRS1041136\_contig\_number\_contig-100\_25085.61951SRS148319\_contig\_number\_contig-100\_5616.5617
1263
genus
7
67

species
1
1262950

SRS023346\_contig\_number\_4650

27
species
41978

SRS011405\_contig\_number\_10368SRS013158\_contig\_number\_contig-100\_3060.30500SRS013687\_contig\_number\_12115SRS014287\_contig\_number\_15629SRS014855\_contig\_number\_9119SRS014948\_contig\_number\_17063SRS015854\_contig\_number\_22181SRS017701\_contig\_number\_10820SRS017916\_contig\_number\_17868SRS018351\_contig\_number\_contig-100\_326.113080SRS018836\_contig\_number\_22298SRS019601\_contig\_number\_29986SRS019968\_contig\_number\_16962SRS023914\_contig\_number\_17982SRS023971\_contig\_number\_4364SRS024009\_contig\_number\_contig-100\_883.39254SRS046369\_contig\_number\_9333SRS050422\_contig\_number\_25169SRS051882\_contig\_number\_19077SRS063985\_contig\_number\_26532SRS075773\_contig\_number\_contig-100\_13586.94847SRS098644\_contig\_number\_26267SRS098655\_contig\_number\_11591SRS140492\_contig\_number\_34102SRS148159\_contig\_number\_9066SRS893341\_contig\_number\_7060SRS893373\_contig\_number\_contig-100\_4004.110906


SRS146813\_contig\_number\_27092
2293195
species
1

14
species
2293179

SRS015190\_contig\_number\_16195SRS017821\_contig\_number\_contig-100\_25.178591SRS018427\_contig\_number\_21264SRS019030\_contig\_number\_17915SRS019381\_contig\_number\_15440SRS043411\_contig\_number\_7773SRS053214\_contig\_number\_7352SRS054956\_contig\_number\_13794SRS058070\_contig\_number\_contig-100\_2979.2979SRS077335\_contig\_number\_contig-100\_80.80SRS077730\_contig\_number\_10126SRS105153\_contig\_number\_26419SRS147919\_contig\_number\_18408SRS148196\_contig\_number\_21249

6
species
165186

SRS020328\_contig\_number\_2598SRS056273\_contig\_number\_26072SRS062701\_contig\_number\_6493SRS077294\_contig\_number\_17806SRS147557\_contig\_number\_33176SRS893252\_contig\_number\_contig-100\_120.102678


SRS020394\_contig\_number\_6022SRS042966\_contig\_number\_1106SRS050925\_contig\_number\_33266SRS077502\_contig\_number\_contig-100\_126.117762SRS1041139\_contig\_number\_366SRS1055056\_contig\_number\_2667SRS144135\_contig\_number\_16710SRS146812\_contig\_number\_49900
2293203
8
species

40518

SRS064757\_contig\_number\_5435SRS1041129\_contig\_number\_15895SRS147653\_contig\_number\_7269
3
species

class
1
909932

1
order
1843489

31977
family
1

1
genus
39948

1955814

SRS014923\_contig\_number\_46653
1
species
